# Design of a High-Efficiency DC-DC Boost Converter for RF Energy Harvesting IoT Sensors

**DOI:** 10.3390/s222410007

**Published:** 2022-12-19

**Authors:** Juntae Kim, Ickjin Kwon

**Affiliations:** Department of Electrical and Computer Engineering, College of Information Technology, Ajou University, Suwon 16499, Republic of Korea

**Keywords:** RF energy harvesting, DC-DC converter, boost converter, high efficiency, efficiency optimization

## Abstract

In this paper, an optimal design of a high-efficiency DC-DC boost converter is proposed for RF energy harvesting Internet of Things (IoT) sensors. Since the output DC voltage of the RF-DC rectifier for RF energy harvesting varies considerably depending on the RF input power, the DC-DC boost converter following the RF-DC rectifier is required to achieve high power conversion efficiency (PCE) in a wide input voltage range. Therefore, based on the loss analysis and modeling of an inductor-based DC-DC boost converter, an optimal design method of design parameters, including inductance and peak inductor current, is proposed to obtain the maximum PCE by minimizing the total loss according to different input voltages in a wide input voltage range. A high-efficiency DC-DC boost converter for RF energy harvesting applications is designed using a 65 nm CMOS process. The modeled total losses agree well with the circuit simulation results and the proposed loss modeling results accurately predict the optimal design parameters to obtain the maximum PCE. Based on the proposed loss modeling, the optimally designed DC-DC boost converter achieves a power conversion efficiency of 96.5% at a low input voltage of 0.1 V and a peak efficiency of 98.4% at an input voltage of 0.4 V.

## 1. Introduction

Energy harvesting technology is considered a key technology for battery-free Internet of Things (IoT) devices [1,2,3]. Since RF signals of various frequency bands such as 5G, Wi-Fi, and TV exist around us, RF energy harvesting can be a very useful energy source [4,5,6,7,8]. However, when the available power of the surrounding RF signal is low, the output DC voltage of the RF rectifier is lower than the voltage required by the system, making it difficult to use as a power source. To increase the DC output voltage, a multi-stage rectifier is required, which significantly reduces the efficiency of energy harvesting [9].

**Figure 1:**
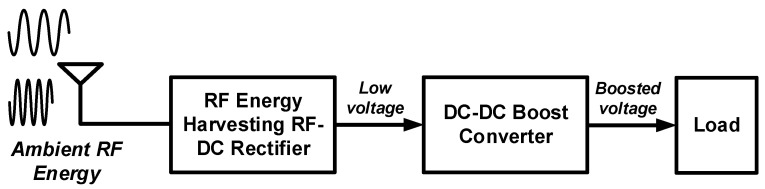
Block diagram of RF energy harvesting system consisting of RF energy harvesting RF-DC rectifier and DC-DC boost converter.

Therefore, the proposed RF energy harvesting system adopts a DC-DC boost converter to convert the low output voltage of the RF-DC rectifier into a high voltage of 1 V or more. Figure 1 shows a block diagram of an RF energy harvesting system consisting of an RF energy harvesting RF-DC rectifier and a DC-DC boost converter. The power management circuit for RF energy harvesting system consists of an RF-DC rectifier that harvests ambient RF energy and converts it to DC voltage, and a DC-DC boost converter that boosts the voltage to a higher DC voltage and supplies it to the load.

### 1.1. Main Contribution

Since the output DC voltage of the RF-DC rectifier for RF energy harvesting varies greatly depending on the RF input power, the DC-DC boost converter is required to achieve high power conversion efficiency (PCE) in a wide input voltage range [4,5,6,7,8,9]. Therefore, to obtain high PCE for different input voltages in a wide input voltage range, an optimal design method of design parameters including an optimal peak inductor current design according to the input voltage is required.

In this paper, we propose an optimal design methodology to achieve high efficiency in a wide input voltage range based on loss analysis and modeling of an inductor-based DC-DC boost converter. In the proposed efficiency optimization design methodology of the DC-DC boost converter, each loss component of the inductor-based DC-DC boost converter is analyzed and the loss modeling result according to the design parameters is presented. Based on the loss analysis and modeling, optimum design parameters including inductance and peak inductor current are obtained to achieve the maximum PCE by minimizing the total loss according to different input voltages in a wide input voltage range.

### 1.2. Organization

This paper is organized as follows. Section 2 introduces the previous DC-DC boost converters for energy harvesting. Section 3 introduces the efficiency problem and the system model of an inductor-based DC-DC boost converter. Section 4 presents loss analysis and modeling of the boost converter. Section 5 focuses on the design methodology for optimizing power conversion efficiency. Section 6 highlights the circuit simulation results. Finally, concluding remarks are given in Section 7.

## 2. Previous DC-DC Boost Converters for Energy Harvesting

DC-DC boost converters based on transformers, switched capacitors, and inductors are used to convert a low input voltage to a high voltage for energy harvesting applications. The transformer-based boost converter operates at a low input voltage of 21 mV without a separate start-up circuit [10], but the transformer increases the area and reduces efficiency at low input voltages. Capacitive-based boost converters are advantageous for on-chip integration because they do not require an inductor and have a relatively small physical form factor [11,12,13,14]. A capacitive-based boost converter with a dynamic body biasing technique that operates at a low input voltage of 0.15 V without a start-up circuit has been demonstrated in [13]. However, it has a limited voltage conversion ratio and relatively low power conversion efficiency at low input voltage. Inductor-based converters are difficult to integrate on-chip due to off-chip inductors but operate at a relatively low input voltage and achieve high power conversion efficiency in a low input voltage range compared with capacitive-based boost converters [15].

In recent works, several approaches have been proposed to improve the power conversion efficiency of low input voltage boost converters for thermoelectric energy harvesting [16,17,18,19,20,21,22,23]. In [17,18], high conversion efficiency is achieved by applying an adaptive gate biasing technique and a peak inductor current control scheme, respectively. However, these designs achieve high efficiency at relatively high input power levels of hundreds of μW or more. In [19], a loss optimization design for MOS switch width and switching frequency has been reported to minimize the total loss to achieve high efficiency in a DC-DC boost converter. In [22], an optimization method for finding the optimum switching frequency, inductance value, and switch size of the converter has been reported. However, these methods obtain high efficiency in a specific input voltage range for thermoelectric energy harvesting, and the efficiency is significantly reduced in a wide input voltage range outside this range.

## 3. Efficiency Problem and System Model of DC-DC Boost Converter

### 3.1. Efficiency Problem in Boost Converter for RF Energy Harvesting

Ambient RF energy harvesting is one of the very useful energy sources for IoT devices, but, when the power of available ambient RF signals is low, the output DC voltage of the RF rectifier is lower than the voltage required by the system, making it difficult to use it as a power source [24,25,26]. Therefore, the proposed RF energy harvesting system adopts a DC-DC boost converter to increase the low output voltage of the RF-DC rectifier to a voltage higher than 1 V. Since the output DC voltage of the RF-DC rectifier for harvesting ambient RF energy varies greatly depending on the RF input power, the voltage applied to the following DC-DC boost converter has a wide input voltage range depending on the ambient RF input power. However, conventional DC-DC boost converters that achieve peak power conversion efficiency (PCE) at a specific input voltage have a problem in that PCE significantly degrades over a wide input voltage range. Therefore, to obtain a high PCE for different input voltages in a wide input voltage range, a design method for optimizing design parameters, including optimal peak inductor current and inductance design according to the input voltage, is required.

### 3.2. System Model of DC-DC Boost Converter

**Figure 2:**
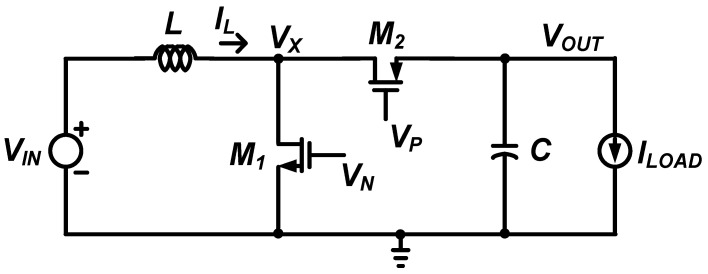
Schematic of an inductor-based DC-DC boost converter.

Figure 2 shows an inductor-based DC-DC boost converter circuit employed to boost the low output DC voltage of the RF rectifier to the high DC voltage required by the circuit in a low power ambient RF energy harvesting operation. The DC-DC boost converter consists of input inductor *L*, NMOS switch *M*_1_, PMOS switch *M*_2_, and a control circuit that drives the switches.

**Figure 3:**
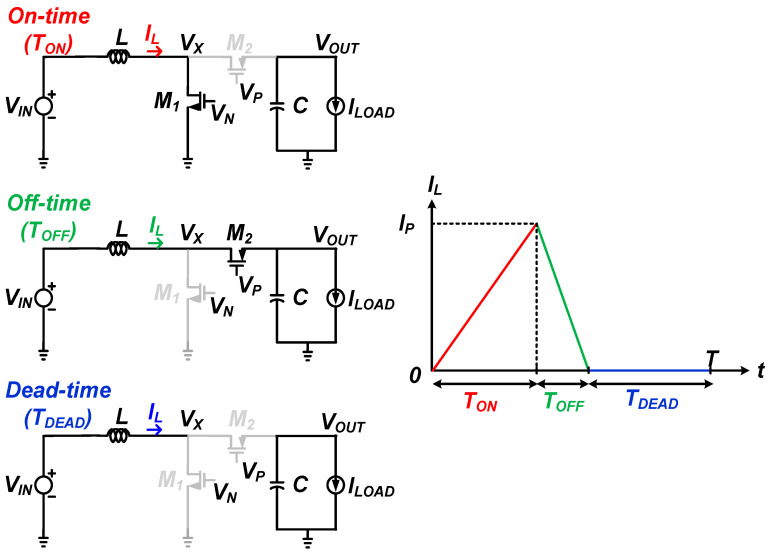
DCM operation and inductor current (*I_L_*) waveform of DC-DC boost converter.

For low input voltage operation, it is more efficient to operate in discontinuous conduction mode (DCM) [21]. Figure 3 shows the DCM operation and inductor current (*I*_L_) waveform of the DC-DC boost converter. The DCM operation of the DC-DC boost converter has three stages: on time (*T*_ON_), off time (*T*_OFF_), and dead time (*T*_DEAD_). During the on-time phase, the NMOS switch *M*_1_ turns on and the PMOS switch *M*_2_ turns off. In this phase, the current through the inductor *I*_L_ increases at a constant slope up to the peak inductor current (*I*_P_), so magnetic energy is stored in the inductor. As the output capacitor *C* is discharged by the constant load current source, the output voltage decreases with a constant slope. During the off-time phase, switch *M*_1_ turns off and switch *M*_2_ turns on. In this phase, the output capacitor is not only discharged by the constant load current but also charged by the current flowing through the inductor. Thus, the energy in the inductor is transferred to the output load current (*I*_LOAD_) and output capacitor, and the inductor current decreases with a constant slope from *I*_P_ to zero. During the dead-time phase, the PMOS switch *M*_2_ opens and the NMOS switch *M*_1_ remains open. In this phase, both the voltage across the inductor and the inductor current flowing through it are zero because both switches are open. The output of the converter is in the same state as the on-time phase, and the output voltage decreases with a constant slope as the output capacitor is discharged by the constant load current source. In DCM operation, the switching frequency is expressed in terms of inductance *L* and peak inductor current *I*_P_ for given *V_IN_*, *V_OUT_*, and *I_LOAD_* conditions as

(1) $$f_{SW}=\frac{2\left(V_{OUT}-V_{IN}\right)I_{LOAD}}{LI_{P}{}^{2}}$$

where *I_LOAD_* is the load current.

In the proposed efficiency optimization design methodology of the DC-DC boost converter, each loss component of the inductor-based DC-DC boost converter is analyzed and the loss modeling result according to the design parameters is presented. Based on the loss analysis and modeling, optimum design parameters that achieve maximum power conversion efficiency by minimizing the total loss according to different input voltages in a wide input voltage range are obtained. The power conversion efficiency (PCE) of the DC-DC boost converter is defined as:

(2) $$\mathrm{PCE}=\frac{P_{OUT}}{P_{IN}}=1-\frac{P_{TOTAL}}{P_{IN}}$$

where *P_IN_* and *P_OUT_* are the input and output power of the converter, respectively, and *P_TOTAL_* represents the total losses of the converter including conduction loss due to equivalent series resistance (ESR) of the inductor, conduction loss due to switches, switching loss of NMOS and PMOS, and switching loss of buffer stages. The proposed design methodology to obtain the maximum PCE by minimizing the total loss according to different input voltages in a wide input voltage range is based on the loss analysis and modeling of an inductor-based DC-DC boost converter.

## 4. Loss Analysis and Modeling of DC-DC Boost Converter

The conduction loss due to the equivalent series resistance (ESR), *R_ESR,L_*, of the inductor is expressed as:

(3) $$P_{COND,DCR}=\frac{1}{3}I_{P}{}^{3}\frac{LR_{ESR,L}}{V_{IN}}f_{SW}+\frac{1}{3}I_{P}{}^{3}\frac{LR_{ESR,L}}{V_{OUT}-V_{IN}}f_{SW}=\frac{2}{3}I_{P}R_{ESR,L}\frac{V_{OUT}}{V_{IN}}I_{LOAD}$$

The conduction loss of the switches is expressed as the sum of the losses due to the on resistance per unit width of the switches *M*_1_ and *M*_2_, *R*_M1_ and *R*_M2_, respectively, and the losses due to the leakage current per unit width of the switches *M*_1_ and *M*_2_, *I_LEAK,M_*_1_ and *I_LEAK,M_*_2_, respectively. The conduction loss due to the switches is expressed as:

(4) $$\begin{array}{lll} P_{COND,SW} & = & \frac{1}{3}I_{P}{}^{3}\frac{L}{V_{IN}}\frac{R_{M1}}{W_{M1}}f_{SW}+\frac{1}{3}I_{P}{}^{3}\frac{L}{V_{OUT}-V_{IN}}\frac{R_{M2}}{W_{M2}}f_{SW}\\ & & +I_{LEAK,M1}W_{M1}V_{IN}+I_{LEAK,M2}W_{M2}\left(V_{OUT}-V_{IN}\right)\\ & = & \frac{2}{3}I_{P}\left(\frac{V_{OUT}-V_{IN}}{V_{IN}}\frac{R_{M1}}{W_{M1}}+\frac{R_{M2}}{W_{M2}}\right)I_{LOAD}\\ & & \;+I_{LEAK,M1}W_{M1}V_{IN}+I_{LEAK,M2}W_{M2}\left(V_{OUT}-V_{IN}\right) \end{array}$$

where *W_M_*_1_ and *W_M_*_2_ are the widths of *M*_1_ and *M*_2_, respectively.

In the DC-DC boost converter, switching losses occur in the transition period by charging and discharging the NMOS and PMOS switch capacitances. The switching loss increases proportionally to the gate–drain capacitances per unit width of *M*_1_ and *M*_2_, *C_GD,M_*_1_ and *C_GD,M_*_2_, respectively, with the Miller effect, the drain–body capacitances per unit width of *M*_1_ and *M*_2_, *C_DB,M_*_1_ and *C_DB,M_*_2_, respectively, and the parasitic capacitance of the inductor, *C_L,PAR_*, expressed as:

(5) $$\begin{array}{lll} P_{SW} & = & \frac{1}{2}f_{SW}V_{OUT}{}^{2}\left[C_{GD,M1}W_{M1}\left(1+\frac{V_{OUT}}{V_{IN}}\right)+C_{DB,M1}W_{M1}\right.\\ & & \left.+C_{GD,M2}W_{M2}\left(1+\frac{V_{OUT}}{V_{IN}}\right)+C_{DB,M2}W_{M2}+C_{L,PAR}\right]\\ & = & \frac{\left(V_{OUT}-V_{IN}\right)I_{LOAD}V_{OUT}{}^{2}}{LI_{P}{}^{2}}\left[C_{GD,M1}W_{M1}\left(1+\frac{V_{OUT}}{V_{IN}}\right)+C_{DB,M1}W_{M1}\right.\\ & & \left.+C_{GD,M2}W_{M2}\left(1+\frac{V_{OUT}}{V_{IN}}\right)+C_{DB,M2}W_{M2}+C_{L,PAR}\right] \end{array}$$

Buffer stages are required as a driver circuit to drive NMOS and PMOS switches. Therefore, in addition to the power consumed to charge and discharge the gate capacitances of the NMOS and PMOS switches, *C_GS,M_*_1_ and *C_GS,M_*_2_, respectively, additional power is consumed by the buffer stages. The switching loss of the buffer stages is proportional to the gate equivalent capacitance of *M*_1_ and *M*_2_ with the Miller effect and is expressed as:

(6) $$\begin{array}{lll} P_{BUFFER} & = & f_{SW}V_{OUT}{}^{2}\left[C_{GS,M1}W_{M1}+C_{GD,M1}W_{M1}\left(1+\frac{V_{IN}}{V_{OUT}}\right)\right.\\ & & \left.+C_{GS,M2}W_{M2}+C_{GD,M2}W_{M2}\left(1+\frac{V_{IN}}{V_{OUT}}\right)\right]\\ & = & \frac{2\left(V_{OUT}-V_{IN}\right)I_{LOAD}V_{OUT}{}^{2}}{LI_{P}{}^{2}}\left[C_{GS,M1}W_{M1}+C_{GD,M1}W_{M1}\left(1+\frac{V_{IN}}{V_{OUT}}\right)\right.\\ & & \left.+C_{GS,M2}W_{M2}+C_{GD,M2}W_{M2}\left(1+\frac{V_{IN}}{V_{OUT}}\right)\right] \end{array}$$

Therefore, the total loss of the DC-DC booster converter including conduction loss due to ESR of the inductor, conduction loss due to switches, switching loss of NMOS and PMOS, and switching loss of buffer stages, respectively, expressed in (3)–(6) is as follows.

(7) $$P_{TOTAL}=P_{COND,DCR}+P_{COND,SW}+P_{SW}+P_{BUFFER}$$

**Figure 4:**
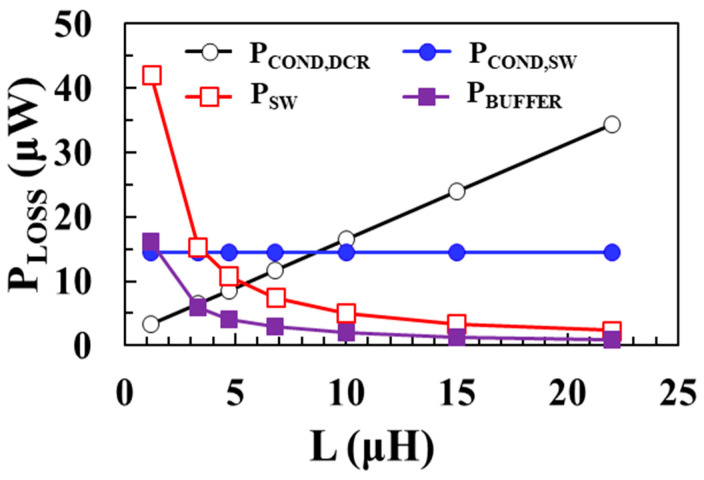
Modeling results of each loss component according to inductance.

Figure 4 shows the modeling results of each loss component of the boost converter according to the inductance *L* using (3)–(6) when *V_IN_* = 0.1 V, *V_OUT_* = 1 V, *I_LOAD_* = 1 mA, *W*_M1_ = *W*_M2_ = 20 mm, and *I_P_* = 30 mA. The inductor is designed as an off-chip component and the DCR of the inductor is modeled as a resistance value that increases with *L*. As shown in Figure 4, as the inductance increases, conduction loss of the inductor increases and switching loss and buffer loss decrease.

**Figure 5:**
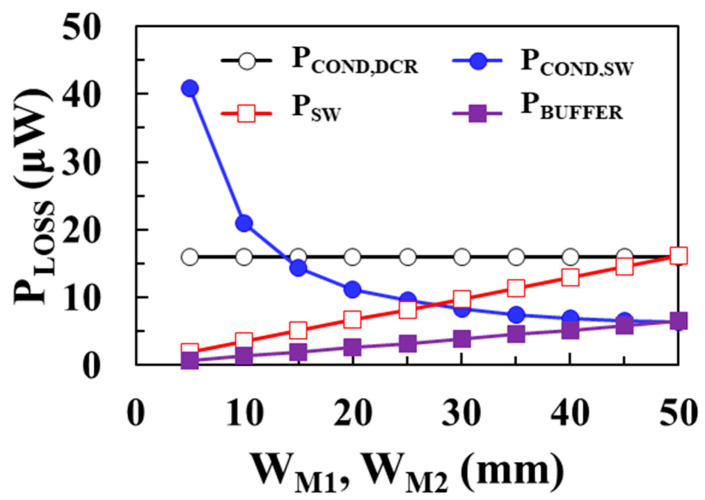
Modeling results of each loss component according to the width of *M*_1_ and *M*_2_ switches.

Figure 5 shows the modeling results of each loss component of the boost converter according to the widths of *M*_1_ and *M*_2_ switches using (3)–(6) when *V*_IN_ = 0.1 V, *V*_OUT_ = 1 V, *I*_LOAD_ = 1 mA, *L* = 10 μH, and *I*_P_ = 30 mA. As shown in Figure 5, as the width of the MOSFET switches increases, conduction loss of the switches decreases and the switching loss and buffer loss increase.

**Figure 6:**
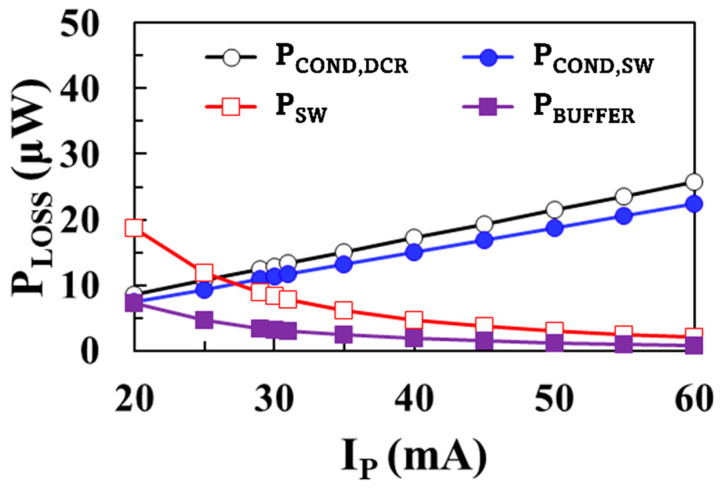
Modeling results of each loss component according to the peak inductor current.

Figure 6 shows the modeling results of each loss component of the boost converter according to the peak inductor current *I*_P_ using (3)–(6) when *V*_IN_ = 0.1 V, *V*_OUT_ = 1 V, *I*_LOAD_ = 1 mA, *W*_M1_ = *W*_M2_ = 20 mm, and *L* = 10 μH. As the peak inductor current increases, conduction loss of the inductor and switches increase and switching loss and buffer loss decrease.

## 5. Proposed Efficiency Optimization Design

**Figure 7:**
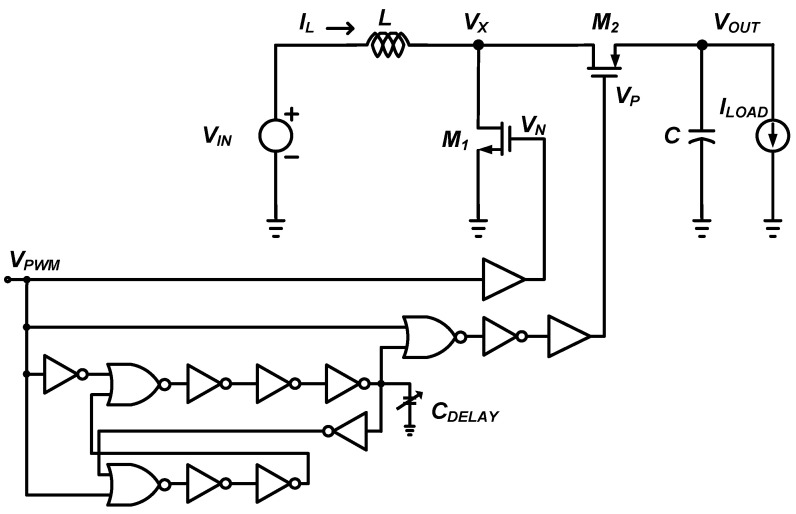
Schematic of the DC-DC boost converter with the control circuits.

Figure 7 shows the schematic of the DC-DC boost converter with the control circuits for generating the pulses to drive and control the switches. The low input voltage DC-DC boost converter operates in discontinuous conduction mode (DCM) to obtain higher efficiency at the low input voltage operation of 0.1 V. A pulse width modulation (PWM) scheme-based control system is applied to keep the output voltage constant in the DC-DC boost converter. The PWM scheme-based control method adjusts the output voltage of the converter under different load current conditions by adjusting the duty cycle of the drive signal with a fixed frequency [18]. The duty cycle of the driving signal to control the NMOS and PMOS switches of a DC-DC converter is proportional to the control voltage, which is the difference between the output voltage of the converter and the reference voltage. The PWM input (*V_PWM_*) is used to drive the switch transistor *M*_1_ through the buffer. The time delay controlled by the digital gates and *C*_DELAY_ compensates for the time delay between the switch control signals *V_N_* and *V_P_* so that transistor *M*_2_ turns on quickly enough after transistor *M*_1_ turns off. The output of the *OR* gate drives the gate of the switch transistor *M*_2_ through a buffer. Current sensing for switch transistor *M*_2_ is performed by a comparator and the comparator’s output signal is used to sense when the current through switch transistor *M*_2_ goes to zero.

In an RF-DC converter for RF energy harvesting, the output DC voltage varies according to the RF input power. Therefore, the DC-DC boost converter following the RF-DC converter is required to achieve high efficiency over a wide input voltage range. To obtain high efficiency in a wide input voltage range, it is necessary to derive the optimal value of each design parameter to minimize the loss by modeling the loss of the converter according to the input voltage.

**Figure 8:**
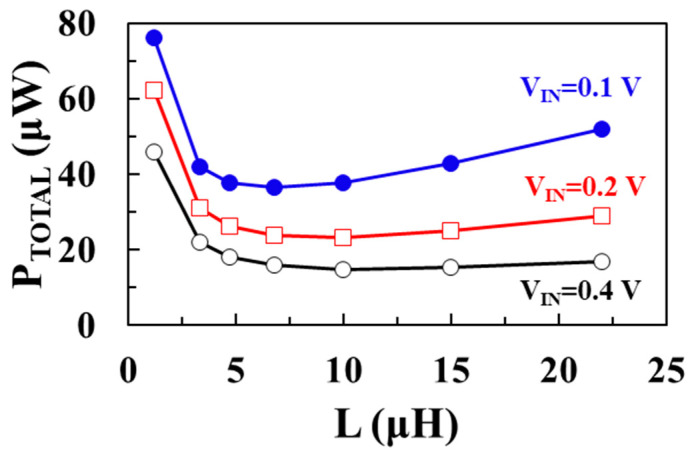
Modeled total loss according to inductance for different input voltages.

**Figure 9:**
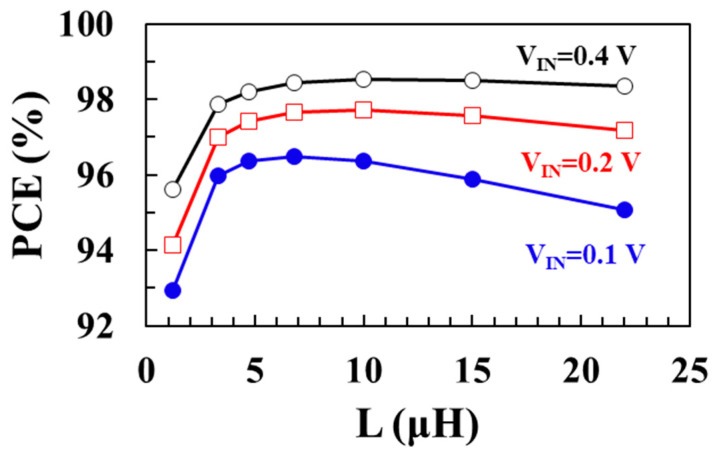
Modeled PCE according to inductance for different input voltages.

Figure 8 shows the modeled total loss (*P_TOTAL_*) as a function of inductance *L* using (7) for input voltages of 0.1 V, 0.2 V, and 0.4 V, respectively, when *V_OUT_* = 1 V, *I_LOAD_* = 1 mA, *W_M_*_1_ = *W_M_*_2_ = 20 mm, and *I_P_* = 30 mA. Figure 9 shows the modeled power conversion efficiency (PCE) as a function of the inductance *L* using (7) for input voltages of 0.1 V, 0.2 V, and 0.4 V, respectively. As the input voltage increases, the optimum inductance to achieve the minimum total loss increases. At an input voltage of 0.1 V, a maximum efficiency of 96.5% is achieved when *L* is 6.8 μH. At input voltages of 0.2 V and 0.4 V, maximum efficiencies of 97.7% and 98.5% are achieved when *L* is 10 μH, respectively.

**Figure 10:**
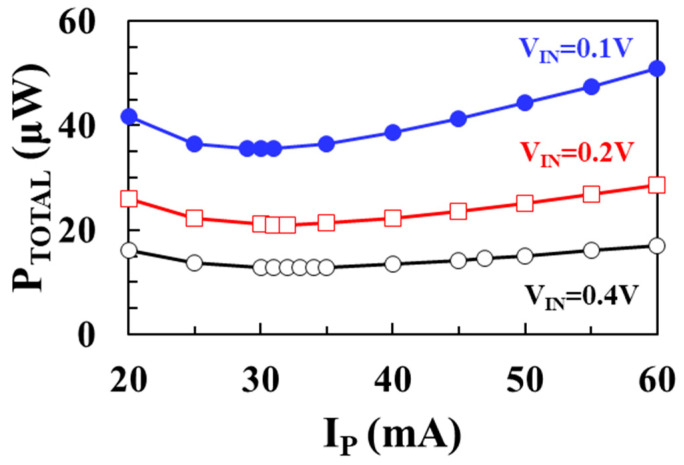
Modeled total loss according to peak inductor current for different input voltages.

**Figure 11:**
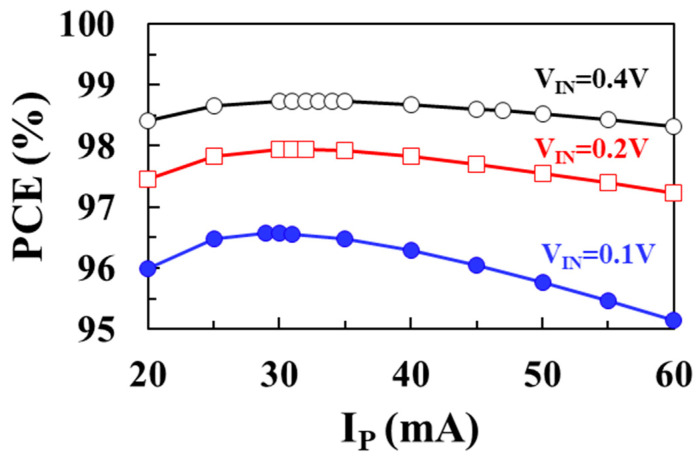
Modeled PCE according to peak inductor current for different input voltages.

Figure 10 shows the modeled total loss (*P_TOTAL_*) as a function of peak inductor current *I*_P_ using (7) for input voltages of 0.1 V, 0.2 V, and 0.4 V, respectively, when *V_OUT_* = 1 V, *I_LOAD_* = 1 mA, *L* = 10 μH, *W_M_*_1_ = *W_M_*_2_ = 20 mm. Figure 11 shows the PCE according to the peak inductor current (*I_P_*) for input voltages of 0.1 V, 0.2 V, and 0.4 V, respectively. As the input voltage increases, the optimum peak inductor current to achieve the minimum total loss increases. At an input voltage of 0.1 V, a maximum efficiency of 96.6% is achieved when *I_P_* is 29 mA. At an input voltage of 0.2 V, a maximum efficiency of 97.7% is achieved when *I_P_* is 31 mA, and at an input voltage of 0.4 V, a maximum efficiency of 98.7% is achieved when *I_P_* is 33 mA.

## 6. Simulation Results and Comparison

**Figure 12:**
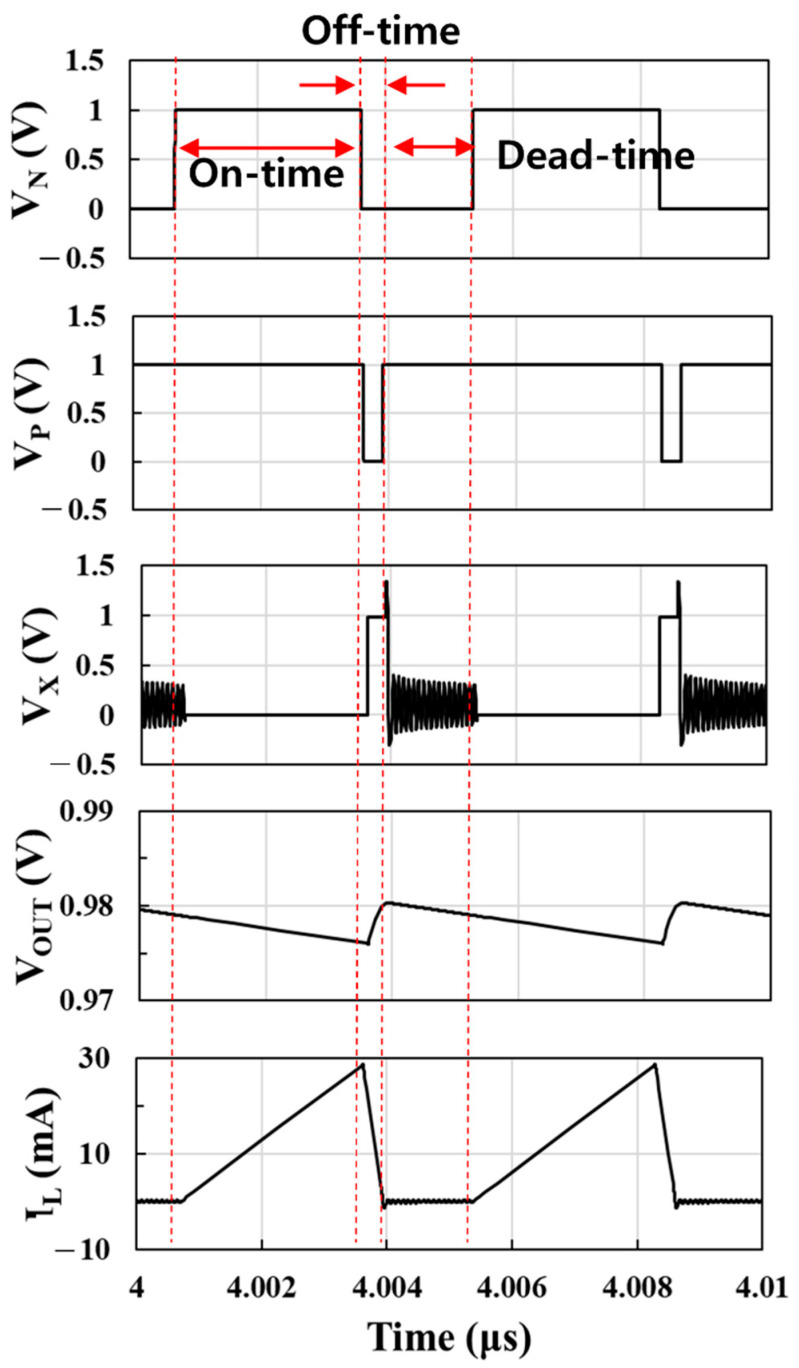
Waveforms of the switch control signals *V_N_* and *V_P_*, internal node voltage *V_X_*, inductor current *I_L_*, and output voltage *V_OUT_* for an input voltage of 0.1 V.

In this paper, the DC-DC boost converter has been designed using a 65 nm CMOS technology. To verify the effectiveness of the DC-DC boost converter and to compare it with the proposed model, post-layout simulations with Spectre were carried out. Figure 12 shows the waveforms of the switch control signals *V_N_* and *V_P_*, internal node voltage *V_X_*, output voltage *V_OUT_*, and inductor current *I_L_* for an input voltage of 0.1 V.

*V_N_* and *V_P_* are the output signals of the control circuit to control the switch on-off of the converter. When *V_N_* and *V_P_* are high at the same time, the inductor is connected to ground during on time so that *I_L_* increases from 0 mA to 29 mA and the load capacitor is discharged by the load current and *V_OUT_* decreases. When *V_N_* and *V_P_* are low at the same time, the inductor is connected to the output during off time and the output capacitor is not only discharged by the constant load current but also charged by the inductor current, so that *V_OUT_* increases. Conduction losses mainly occur as current flows during on time and off time. During the dead time when *V_N_* is low and *V_P_* is high, no current flows through the inductor and, as in the on-time period, the output capacitor is discharged by constant load current and *V_OUT_* decreases. Ringing occurs due to the resonance of the inductor and parasitic capacitor during dead time.

**Figure 13:**
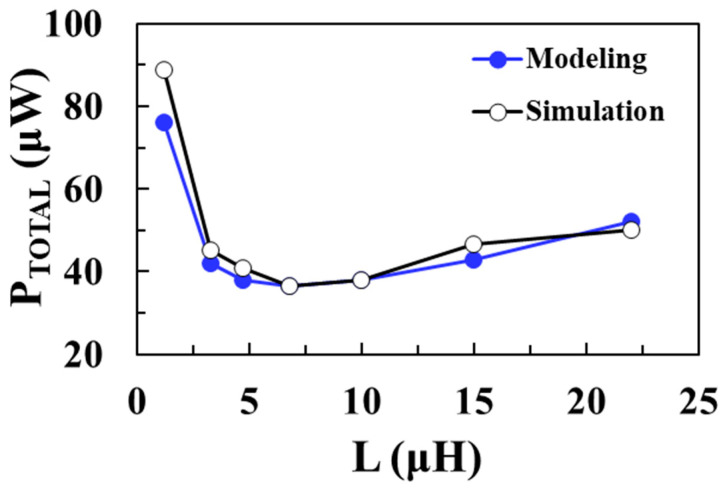
Total loss according to inductance for an input voltage of 0.1 V and comparison with the modeling results.

**Figure 14:**
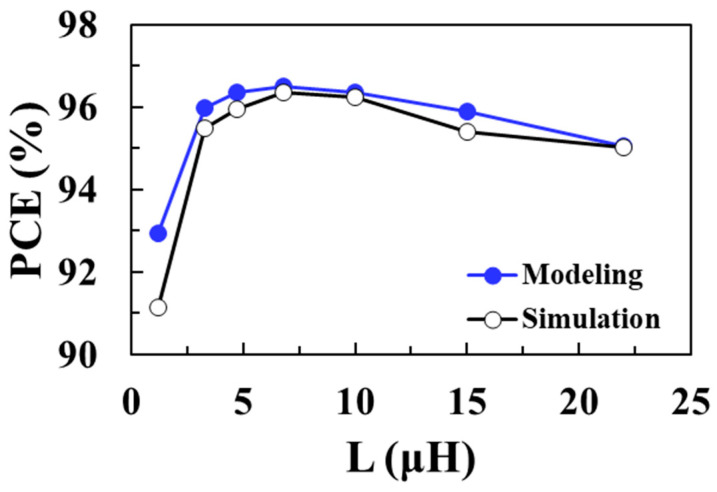
PCE according to inductance for an input voltage of 0.1 V and comparison with the modeling results.

Figure 13 shows the total loss of the DC-DC boost converter according to the inductance *L* for an input voltage of 0.1 V and compares the circuit simulation results with the proposed modeling results when *V_OUT_* = 1 V, *I_LOAD_* = 1 mA, *W_M_*_1_ = *W_M_*_2_ = 20 mm, and *I_P_* = 30 mA. As the inductance decreases, the switching frequency increases and the switching losses of NMOS and PMOS increase. On the other hand, as the inductance increases, the ESR of the inductor increases and conduction loss of the inductor increases. Therefore, there is an optimal inductance value that minimizes the total loss. It shows that the modeled total loss of the boost converter agrees well with the circuit simulation results, with a minimum loss of 36.4 μW achieved for an *L* of 6.8 μH. Figure 14 shows the PCE of the converter according to *L* for an input voltage of 0.1 V and compares the circuit simulation results with the proposed modeling results. A maximum efficiency of 96.5% is achieved at an optimum *L* of 6.8 μH and the proposed model accurately predicts the optimal *L* value to obtain the maximum PCE.

**Figure 15:**
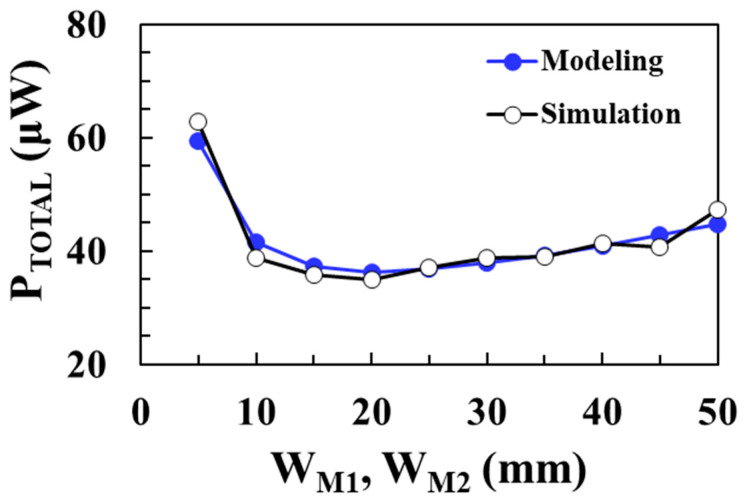
Total loss according to the switch width for an input voltage of 0.1 V and comparison with the modeling results.

**Figure 16:**
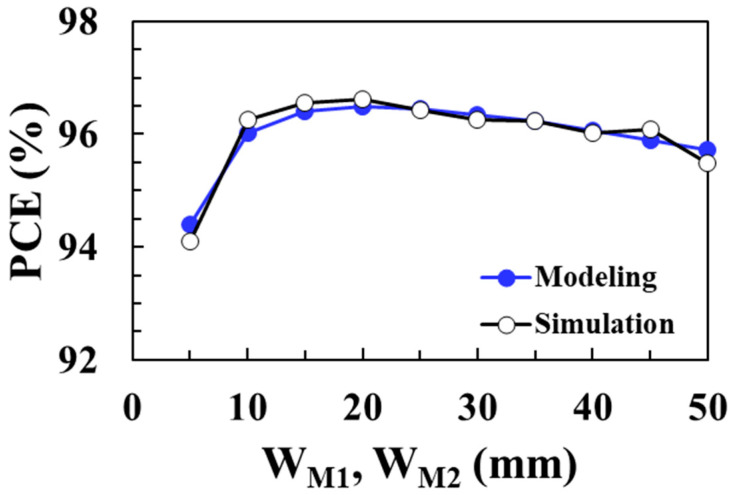
PCE according to switch width for an input voltage of 0.1 V and comparison with the modeling results.

Figure 15 shows the total loss of the DC-DC boost converter according to the width of *M*_1_ and *M*_2_ switches for an input voltage of 0.1 V and compares the circuit simulation results with the proposed modeling results when *V_OUT_* = 1 V, *I_LOAD_* = 1 mA, *L* = 10 μH, and *I*_P_ = 30 mA. As the NMOS and PMOS switches’ width decreases, the on resistance increases and the conduction losses of the switches increase. On the other hand, as the switches’ width increases, the capacitances increase and the switching losses of the NMOS and PMOS increase. Therefore, there is an optimal switch width that minimizes the total loss. The modeled total loss agrees well with the circuit simulation results and a minimum loss of 36.3 μW is achieved when the switch width is 20 mm. Figure 16 shows the PCE of the boost converter according to the width of *M*_1_ and *M*_2_ switches for an input voltage of 0.1 V and compares the circuit simulation results with the proposed modeling results. A maximum efficiency of 96.5% is achieved at an optimum switch width of 20 mm and the proposed model accurately predicts the optimal switch width to obtain the maximum PCE.

**Figure 17:**
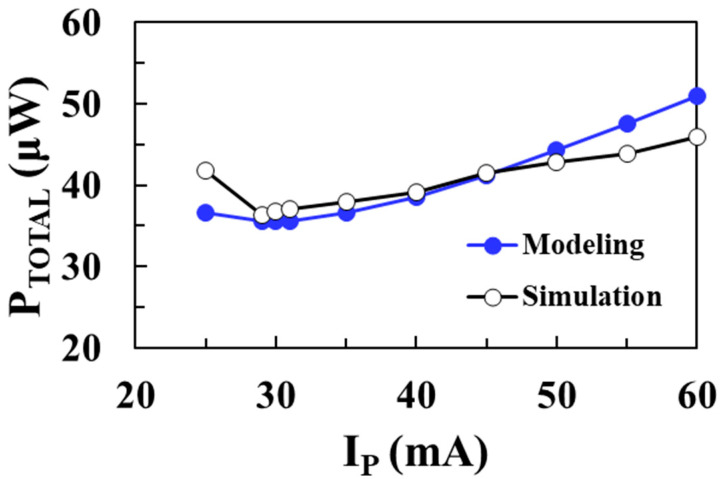
Total loss according to the peak inductor current for an input voltage of 0.1 V and comparison with the modeling results.

**Figure 18:**
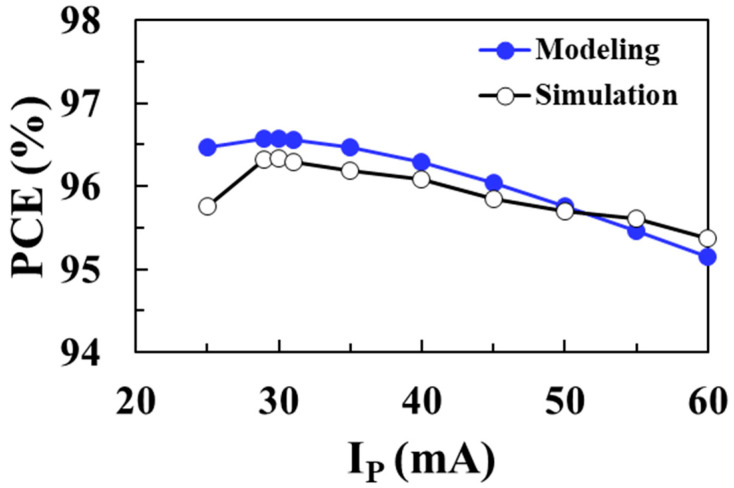
PCE according to the peak inductor current for an input voltage of 0.1 V and comparison with the modeling results.

Figure 17 shows the total loss of the DC-DC boost converter according to the peak inductor current *I*_P_ for an input voltage of 0.1 V and compares the circuit simulation results with the proposed modeling results when *V_OUT_* = 1 V, *I_LOAD_* = 1 mA, *L* = 10 μH, and *W_M_*_1_ = *W_M_*_2_ = 20 mm. As the peak inductor current decreases, the switching frequency increases and the switching losses of the NMOS and PMOS increase. On the other hand, as the peak inductor current increases, conduction losses in the switches and inductor increase. Therefore, there is an optimal peak inductor current that minimizes total losses. The modeled total loss agrees well with the circuit simulation results, with a minimum loss of 35.6 μW achieved at an *I_P_* of 29 mA. Figure 18 shows the PCE of the boost converter according to the peak inductor current *I*_P_ for an input voltage of 0.1 V and compares the circuit simulation results with the proposed modeling results. A maximum efficiency of 96.6% is achieved at an optimum *I_P_* of 29 mA and the proposed model accurately predicts the optimal *I_P_* to obtain the maximum PCE.

**Figure 19:**
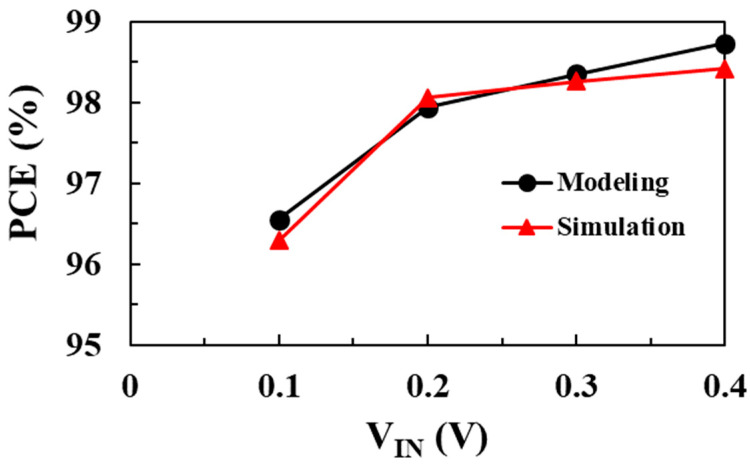
PCE according to input voltage comparison with the modeling results.

Figure 19 shows the PCE of the converter according to the input voltage and compares the circuit simulation results with the proposed modeling results when *V_OUT_* = 1 V, *I_LOAD_* = 1 mA, and *W_M_*_1_ = *W_M2_* = 20 mm. Based on the proposed optimal design methodology to achieve the maximum PCE, the optimal values of inductance and peak inductor current are designed differently according to the input voltage. As the input voltage increases, the optimum inductance and peak inductor current to achieve the minimum total loss increase. At an input voltage of 0.1 V, a maximum PCE of 96.5% is achieved when *L* is 6.8 μH and *I*_P_ is 29 mA. On the other hand, at an input voltage of 0.4 V, a maximum PCE of 98.4% is achieved when *L* is 10 μH and *I_P_* is 33 mA. The PCE modeling results achieved from the proposed efficiency optimization design method based on loss analysis and modeling are in good agreement with circuit simulation results over a wide input voltage range. By deriving the optimal design parameters including inductance and peak inductor current for different input voltages, the maximum PCE is achieved over a wide input voltage range.

Table 1 summarizes the performance of this work and compares with the state-of-the-art DC-DC boost converters for energy harvesting applications. The proposed boost converter for RF energy harvesting operates over a wide input voltage range and achieves a PCE of 96.5% at a low input voltage of 0.1 V and the highest peak efficiency of 98.4% at an input voltage of 0.4 V.

## 7. Conclusions

In this paper, an optimization design methodology for a high-efficiency DC-DC boost converter is proposed based on loss analysis and modeling of an inductor-based DC-DC boost converter. In the proposed efficiency optimization design methodology of the DC-DC boost converter, each loss component of the inductor-based DC-DC boost converter is analyzed and the loss modeling result according to the design parameters is presented. Based on the loss analysis and modeling, optimum design parameters including inductance and peak inductor current are obtained to achieve the maximum PCE by minimizing the total loss according to different input voltages in a wide input voltage range. The modeled total losses agree well with the circuit simulation results and the proposed loss modeling results accurately predict the optimum design parameters to obtain the maximum PCE. The designed DC-DC boost converter achieves a power conversion efficiency of 96.5% at a low input voltage of 0.1 V and a peak efficiency of 98.4% at an input voltage of 0.4 V. To further improve the power conversion efficiency of the DC-DC boost converter for RF energy harvesting, not only the efficiency optimization design method of the proposed converter core circuit but also the efficiency optimization design method of the converter switch control circuit is required; therefore, further research on this is needed in the future.

## Figures and Tables

**Table 1:** Performance summary and comparison.

Reference	[18]	[19]	[20]	[23]	This Work
CMOS technology	130 nm	180 nm	65 nm	180 nm	65 nm
Output voltage	1.1 V	0.8–1.1 V	1–1.1 V	0.9–1.4 V	1 V
Load current	0.22 μA	–	–	–	0.1–1 mA
Inductance	10 μH	47 μH	10 μH	47 μH	6.8 μH @ 0.1 V 10 μH @ 0.4 V
PCE @ Min. *V*_IN_	30% @ 0.05 V	48% @ 0.02 V	10% @ 0.04V	60% @ 0.02 V	96.5% @ 0.1 V
Max. PCE @ *V*_IN_	83% @ 0.3 V	53% @ 0.06 V	75% @ 0.2 V	90.8% @ 0.18 V	98.4% @ 0.4 V

## Data Availability

Not applicable.

## Abbreviations

**Symbol/Notation**	**Description**
*f_SW_*	switching frequency
*I_P_*	peak inductor current
*I_LOAD_*	load current
*L*	inductance
*W_M1_ and W_M_*_2_	width of the switches *M*_1_ and *M*_2_
PCE	power conversion efficiency
*P_TOTAL_*	total loss of the converter
*P_COND,DCR_*	conduction loss due to the resistance of inductor
*P_COND,SW_*	conduction loss due to the switches
*P_SW_*	switching loss of the NMOS and PMOS switches
*P_BUFFER_*	switching loss of the buffer stages
